# Properties of Soil Pore Space Regulate Pathways of Plant Residue Decomposition and Community Structure of Associated Bacteria

**DOI:** 10.1371/journal.pone.0123999

**Published:** 2015-04-24

**Authors:** Wakene C. Negassa, Andrey K. Guber, Alexandra N. Kravchenko, Terence L. Marsh, Britton Hildebrandt, Mark L. Rivers

**Affiliations:** 1 IASS-Global Soil Forum, Institute for Advanced Sustainability Studies, Potsdam, Germany; 2 Department of Plant, Soil and Microbial Sciences, Michigan State University, East Lansing, Michigan, United States of America; 3 Department of Microbiology and Molecular Genetics, Michigan State University, East Lansing, Michigan, United States of America; 4 Center for Advanced Radiation Sources, The University of Chicago, Argonne National Lab, Argonne, Illinois, United States of America; Chinese Academy of Sciences, CHINA

## Abstract

Physical protection of soil carbon (C) is one of the important components of C storage. However, its exact mechanisms are still not sufficiently lucid. The goal of this study was to explore the influence of soil structure, that is, soil pore spatial arrangements, with and without presence of plant residue on (i) decomposition of added plant residue, (ii) CO_2_ emission from soil, and (iii) structure of soil bacterial communities. The study consisted of several soil incubation experiments with samples of contrasting pore characteristics with/without plant residue, accompanied by X-ray micro-tomographic analyses of soil pores and by microbial community analysis of amplified 16S–18S rRNA genes via pyrosequencing. We observed that in the samples with substantial presence of air-filled well-connected large (>30 µm) pores, 75–80% of the added plant residue was decomposed, cumulative CO_2_ emission constituted 1,200 µm C g^-1^ soil, and movement of C from decomposing plant residue into adjacent soil was insignificant. In the samples with greater abundance of water-filled small pores, 60% of the added plant residue was decomposed, cumulative CO_2_ emission constituted 2,000 µm C g^-1^ soil, and the movement of residue C into adjacent soil was substantial. In the absence of plant residue the influence of pore characteristics on CO_2_ emission, that is on decomposition of the native soil organic C, was negligible. The microbial communities on the plant residue in the samples with large pores had more microbial groups known to be cellulose decomposers, that is, Bacteroidetes, Proteobacteria, Actinobacteria, and Firmicutes, while a number of oligotrophic Acidobacteria groups were more abundant on the plant residue from the samples with small pores. This study provides the first experimental evidence that characteristics of soil pores and their air/water flow status determine the phylogenetic composition of the local microbial community and directions and magnitudes of soil C decomposition processes.

## Introduction

Soil is a critical component of terrestrial ecosystems, harboring enormous microbial diversity, moderating the decomposition cycle and greatly influencing atmospheric CO_2_ concentrations [1,2]. Soil organic matter (SOM) contains more than twice the amount of carbon (C) than all terrestrial biota [3] and increasing the levels of soil organic C by, in part, decreasing decomposition of SOM can be a feasible means for reducing atmospheric CO_2_ levels [4]. Soil structure defines sizes and characteristics of soil pores, thus creating physical micro-environments for microbial activities and playing a major role as both an arena and a product of SOM stabilization and dynamics [5–8]. Microorganisms are vital actors of soil systems. Their ability to move and grow in a structurally and chemically heterogeneous soil environment governs belowground processes of biomass transformation into SOM and defines its further fate, e.g., stabilization within soil matrix or loss to the atmosphere as emitted CO_2_ [9]. Distribution of microorganisms and their activity in soil is spatially correlated at scales ranging from microns to centimeters [10–12]. The spatial patterns in distributions of microorganisms are related to presence and characteristics of soil pores [13–15]. Pores control micro-scale soil air, water, and nutrient fluxes; thus, influence micro-environmental conditions for microbial functioning, e.g., [16–18]. It has been shown that micro-environmental conditions may exert even a stronger influence on SOM decomposition than microbial community composition [19].

Substantial efforts have been invested by the scientific community into understanding empirical relationships between soil structure and soil C [20–26]. These efforts have greatly increased our conceptual vision of soil C sequestration and the crucial importance of soil structure in it, e.g., [3]). It is generally accepted that physical protection of organic C in undisturbed soil is achieved due to those characteristics of soil structure that may limit the ability of microbes to reach it, e.g., [27–30]. However, mechanisms behind the interactions between soil structure, the spatial patterns of organic C distribution, and ability of microbes to reach and decompose it are still not sufficiently lucid to enable deterministic modeling and accurate predictions.

Surprisingly, results of multiple attempts of linking micro-scale information regarding soil pores with soil C processes remained inconclusive. Specifically, very few studies found relationships between decomposition of native SOM and soil pores, even in the studies specifically designed to create contrasting pore characteristics [31, 32]. A possible explanation proposed by Juarez et al. [32] is that native C decomposition is controlled by processes taking place at a scale below the one at which the pore structures are manipulated; possibly in pores <1 μm in size or at surfaces of soil particles.

On the contrary, associations between pores and decomposition rates were observed in the studies where fresh plant residues were added to soil samples with contrasting pore characteristics [33–35]. In the latter cases, not only was the decomposition of plant residues influenced by pore characteristics, but the presence of decomposing residues itself led to formation of new pores. This discrepancy suggests that soil pore characteristics do influence soil C processes. However, the magnitudes and rates of such influences are moderated by presence/absence of plant residues.

The decomposition of plant residues freshly added to soil often leads to increased decomposition of native SOM, a phenomenon known as priming effect, e.g., [36], and the increases in mineralization of native soil C due to plant residue additions can be substantial [37,38]. Priming effect is believed to be one of the major risk factors in increasing susceptibility of SOM to decomposition under changing climate [39, 40]. However, quantitative assessment of the contribution of plant residue to soil C processes remains inadequate [19]. A major deterrent is a lack of understanding of the role that physical protection mechanisms, e.g., soil pores, play in the interactions between plant residue presence and soil processes, including water and air movements and microbial activities.

Application of X-ray computed micro-tomography (μCT) tools offers new promise for gaining not just a better qualitative understanding but for developing quantitative descriptions of the relationships between soil C processes and physical environments that enable C protection at micron resolution [41–46]. X-ray μCT enables visualization of pores within intact soil samples and identification of pore sizes, connectivities, and other characteristics necessary to describe air and water fluxes within the samples [44,46–48]. X-ray μCT images can provide information on location and characteristics of large pieces of organic material, including plant roots, plant residues, and particulate organic matter, e.g., [26, 33, 49, 50], thus enabling monitoring of their *in situ* decomposition.

The goal of this study is to take advantage of the latest advances in X-ray μCT tools to explore interactions between the presence of plant residue and the soil structure, as represented by characteristics of soil pores, in their influence on soil C cycling. Consistent with previous findings, we expect that plant residue inputs will have a major influence on soil C cycling. However, we hypothesize that it is the soil pore characteristics and their influence on micro-scale air and water distributions that select the composition of the microbial communities and determine the rates and magnitudes of decomposition of the added plant residue. These activities in concert then define the decomposition of native soil C. The specific objectives of the study are to explore how the interactions between soil pore characteristics and plant residue affect 1) plant residue decomposition, 2) CO_2_ emission from soil samples, and 3) composition of bacterial communities associated with the plant residue.

## Materials and Methods

### Ethics statement

Permission for collecting soil samples has been obtained from the Executive Committee of the Long Term Ecological Research site experiment at W. K. Kellogg Biological Station (LTER KBS). Permission was obtained upon submission of the site use request form (http://lter.kbs.msu.edu/research/conducting-research/). The samples did not involve any endangered species.

### Site description and soil sampling

Soil for the study was collected at conventionally managed chisel-plowed corn-soybean-wheat rotation system of LTER KBS, in southwest Michigan, USA (85°24' W, 42°24' N). The soil is fine-loamy, mixed, mesic Typic Hapludalf (Kalamazoo series) developed on glacial outwash [51]. Three blocks of soil samples approximately 15 cm x 15 cm x 15 cm in size were collected from 0–15 cm depth with a spade in February of 2012. The samples were air dried, crushed, composited and manually sieved to <2.0 mm size. About 50 g of the <2 mm composite sample was placed on a nest of sieves and mechanically sieved with RO-TAP test sieve shaker (Model RX-29, OH, USA) for one minute to obtain soil aggregate fractions of five different sizes: <0.05, 0.05–0.10, 0.10–0.50, 0.50–1.00, and 1.00–2.00 mm. The aggregate fractions were used for the incubation experiments, the μCT scanning, and the analyses of selected basic soil properties.

### Analysis of basic soil properties

Selected basic soil properties were determined from the studied soil aggregate fractions in six replications. Total C and N were measured with the elemental combustion system (ECS 4010 Costech Analytical, USA) [52]. Soil pH was determined at 1:1 soil: deionized water ratio [53].

### Incubation experiment setup

Soil samples with contrasting pore characteristics were subjected to incubation with and without presence of plant residue, along with determination of bacterial community structure. X-ray μCT was used to assess soil pore characteristics and to quantify decomposition of the added plant residue. The outline of the study’s experiments is summarized in the paragraph below with specific details provided further in this section.

Five soil aggregate fractions, i.e., <0.05, 0.05–0.10, 0.10–0.50, 0.50–1.00, and 1.00–2.00 mm, were used for the incubation experiments (S1 Table). For each aggregate fraction size we created two types of samples: intact and ground. For each sample type of each fraction size we incubated the samples with and without added plant residue. Both intact and ground samples with added plant residue were subjected to μCT scanning. For the analyses of bacterial communities we used samples of the two aggregate fractions that exhibited most contrasting CO_2_ emission and plant residue decomposition results. These fractions were 0.05–0.10 mm and 1.00–2.00 mm. Both intact and ground samples of these two fractions with added plant residue were used in microbial community analyses.

Each incubation sample consisted of approximately 0.6 g of air-dry soil. For incubation the samples were placed in 3 ml plastic syringes (Ø 10 mm) (BD Franklin Lakes NJ, USA) after covering the base of the syringe with glass wool. Plant residue that was used in the study consisted of round pieces (Ø 8 mm) cut from dried corn leaves using Realeather Crafts Maxi Puncher Set. The average weight of the cut leaf pieces used in the study was approximately 2.5 mg. The average concentrations of total C and N in the leaves were 387 and 30 mg g^-1^ leaf, respectively.

To prepare the samples amended with plant residue, each soil sample was subdivided into two halves, ~0.3 g each. One half was placed in the syringe, the corn leaf piece was placed horizontally on top of it and then covered with the rest of the soil. These samples are referred to as samples with leaves. A schematic representation of the steps involved in assembling the samples with leaves for the incubation experiment is shown in S1 Fig Samples without plant residue were prepared by placing the entire amounts of soil into the syringes. These samples are referred to as samples without leaves.

For the incubation experiment, soil water content in all samples was maintained close to 50% of the total soil porosity. To ensure a uniform soil water distribution, half of the water was added from the bottom and the other half from the top of the sample (S1 Fig). In the samples without leaves the entire amount of water was added from the top of the sample. After adding water, the base of each syringe was covered with rubber sleeve stopper, and each syringe was placed in a 10 ml vacutainer (BD Franklin Lakes NJ, USA). Approximately 0.5 ml of water was added to the bottom of each vacutainer to protect soil from drying during the incubation.

Of particular importance for our study was to estimate the differences in CO_2_ emission and plant residue decomposition that were due to contrasting pore characteristics. To achieve this goal such differences had to be separated from the differences originated from potential dissimilarities in chemical or biological characteristics of different aggregate fractions. The purpose of ground samples was to enable us to separate the differences related to pore characteristics. Ground samples of each of the studied aggregate size fractions were prepared by grinding soil of the fraction in a shatter box grinder (Shatter Box 8530, USA) to pass a 0.05 mm sieve. Then samples with and without leaves were prepared and were subjected to incubation using the same procedures as for the soil samples of intact aggregate fractions.

For the incubation experiments, we used six replications for intact soil aggregate fractions with leaves, and four replications for intact samples without leaves. Five replications of ground soil aggregate fractions with and without leaves were used.

The incubations were carried out at 22^o^ C for 120 days. The CO_2_ emission was measured on the first, second, fourth and eighth days of the incubation and then continued on a weekly basis until the last month of the study, when CO_2_ was measured at two and three week intervals. The CO_2_ measurements were conducted using infrared gas analyzer (LI-820 CO_2_ Analyzer Lincoln, Nebraska, USA). After each sampling, the remaining gas in the headspace was flushed with CO_2_-free air.

After completing the incubations and scanning, the intact and ground samples with leaves were disassembled, and approximately 3–4 mm of soil material adjacent to the leaves was used to measure soil total C.

### μCT Scanning and image analysis

Two replications of the intact samples of all five fractions with leaves were subjected to μCT scanning before and after the incubation. Four remaining replications of the intact samples with leaves and all five replications of the ground samples with leaves of the <0.05, 0.05–0.1 and 1.0–2.0 mm size fractions were μCT scanned after the incubation. These three fractions were selected for after-incubation scanning because they produced most contrasting CO_2_ emission and plant residue decomposition results. All samples were air-dried prior to scanning.

Scanning was conducted on the bending magnet beam line, station 13-BM-D of the GeoSoilEnvironCARS (GSECARS) at the Advanced Photon Source (APS), Argonne National Laboratory (ANL), IL. Data were collected with the Si (111) double crystal monochromator tuned to 28 keV incident energy, the distance from the sample to the source was approximately 55 m. 2D projections were taken at 0.25^°^ rotation angle steps and combined into a 3D image consisting of 1040 slices with 1392x1392 pixels per slice. The scanning resolution was 6.5 μm. Detailed information on the procedure and data preprocessing steps is given in [54] and [55].

Classification of image voxels into pore or solid material was conducted using the indicator kriging method [56, 57]. Four pore characteristics were obtained from the images, namely, total porosity, image-based porosity, size distributions of >6.5 μm pores, and connectivity of >6.5 μm pores. Total porosity was determined from dry weight and volume values of each soil sample. Image-based porosity was assessed as the percent of pores visible at the image resolution (>6.5 μm). Pore-size distributions were obtained via burn number distribution approach using 3DMA-Rock software [47] and the details of its implementation are described in Wang et al. [46]. Connectivity of pores visible at the image resolution (>6.5 μm) was determined using SCAMP V1.2 (developed in SIMBIOS Centre, University of Abertay, Dundee, Scotland) implemented in ImageJ [58].

Pieces of leaves were identified on the images using a combination of tools available from ImageJ along with its plug-in tools 3D Viewer [59] and BoneJ [60], and the techniques used in the approach for particulate organic matter determination developed by Kravchenko et al. [61]. Specifically, leaf identification started from preliminary selection of leaf voxels based on their grayscale values. That was followed by maximum filtering and fine-tuning the selection based on the grayscale values of the filtered image. Then a series of noise reduction and image erosion steps was applied in order to remove the features from the rest of the soil image that happened to have the same range of grayscale values as the leaf. Identified leaf images were then visually inspected and misclassified elements, e.g., soil particles closely aligned with the leaf, were removed manually. The volume of the leaf was obtained as a product of the number of leaf voxels and the voxel volume (6.5 x 6.5 x 6.5 μm^3^).

For the samples that were scanned both before and after the incubation, the amount of leaf material lost during the incubation was determined as the difference between leaf volumes measured before and after incubation. The average initial leaf volume was calculated from the data on all the samples scanned prior to the incubation (n = 6). Then for the samples that were scanned only after the incubation the average initial leaf volume was used as the estimate of the before-incubation leaf volume.

We used the information on pore presence and their water-filled status to estimate gas diffusion coefficients using the approach proposed by Resurreccion et al. [62]. The approach is based on the conceptual model of a bimodal porous medium with pore space divided into inter- and intra-aggregate pore regions with the region specific values of the gas diffusion coefficients. Since soil samples of this study were constructed as mixtures of aggregates of different sizes, the approach of Resurreccion et al. [62] appeared to be particularly suitable for gas diffusivity estimation in our samples. These calculations were performed for illustration purpose only for the two contrasting intact aggregate samples of 0.05–0.1 mm and 1–2 mm sizes.

Resurreccion et al. [62] showed that the ratio between the gas diffusion coefficients in the soil, *D*_*p*_, and in the free air, *D*_*o*_, changes with water content as:

$$\frac{D_{p}}{D_{0}}=\{\begin{array}{c} {\left(\frac{\varepsilon_{1}}{\Phi_{1}}\right)}^{N_{1}}\varepsilon_{1}{}^{X_{1}}\;\varepsilon \leq \Phi_{\text{1}}\\ \Phi_{1}^{X_{1}}+F_{2}\varepsilon_{2}^{X_{2}}\;\varepsilon >\Phi_{\text{1}} \end{array}$$ (1)

where Φ_1_ and Φ_2_ are the inter- and intra-aggregate air-filled porosities, respectively, (cm^3^ cm^-3^); *ε*_1_, *ε*_2_ and *ε* are the inter- aggregate, intra-aggregate, and total air-filled porosities, respectively, (cm^3^ cm^-3^); *X*_1_ and *X*_2_ are the dry-region pore connectivity factors of inter- and intra-aggregate pores, (dimensionless); and *N*_1_ and *F*_2_ are the empirical parameters, (dimensionless).

In the gas diffusion estimation for the 1–2 mm aggregate fraction we used parameters obtained by Resurreccion et al. [62] for the 1–2 mm aggregate fraction of a silty clay soil [63]. We used 50% air-filled porosity, consistent with our experimental settings; and based on the image analyses we estimated that approximately 65% of the pores were in the inter-aggregate air-filled group. In the gas diffusion estimation for the 0.05–0.1 mm aggregate fraction the (Eq 1) was applied using condition *ε≤*Φ_1_ with Φ_1_ = Φ assuming absence of the intra-aggregate pores in this size fraction.

### Microbial Analyses

To assess the microbial community composition on the decomposing leaves we prepared 5 replicates of the intact and ground samples with leaves for two aggregate fractions with the most contrasting pore characteristics, CO_2_ emission, and plant residue decomposition results, i.e. 0.05–0.1 mm and 1–2 mm fractions. The samples for microbial community analysis were prepared and incubated as described above. However, the incubation was conducted only for 14 days. After 14 days, the leaves were removed from the samples and subjected to microbial analyses. Shorter incubation time was used because of concerns regarding reduction in microbial activities and changes in microbial communities during long-term incubation. The first 1–3 weeks are known as the time when residue decomposition is most vigorous [64, 65], therefore our microbial community analysis aimed at capturing the communities present in the samples during active residue decomposition stage.

#### DNA extraction, amplification, and pyrosequencing

DNA was extracted from the leaf samples, ranging from 20 mg to 70 mg, using the PowerSoil DNA Isolation Kit (Mo Bio Laboratories Inc., Carlsbad, CA), according to the manufacturer’s instructions. Extracted DNA was stored at -20°C until needed. The V3-V5 region of the 16S rRNA gene was targeted for pyrosequencing using HMP primers (357F and 926R). The 357F primer included the 454 adapter sequence and the primer sequence 5’-CCTACGGGAGGC AGCAG-3’. The 926R primer contained the 454 adapter sequence, unique barcodes and the sequence 5’-CCGTCAATTCMTTTRAGT-3’. PCR amplifications were performed in duplicate. Each 75 μL PCR reaction contained 5 μL of template (2–7 ng μL^-1^), 3.0 U of AccuPrime Taq DNA Polymerase High Fidelity (Invitrogen Corp., Carlsbad, CA), 60 mM Tris-SO4 (pH 8.9), 18 mM (NH_4_)_2_SO_4_, 2 mM MgSO_4_, 0.2 mM of each deoxynucleoside triphosphate, and 0.2 μM of each primer. PCR was performed under the following cycle conditions: an initial denaturation step at 98°C for 30 s and 25 cycles of denaturation at 94°C for 1 min, annealing at 56°C for 45 s, and extension at 72°C for 100 s. A final extension step at 72°C for 8 min was then performed. Gel analysis was performed to confirm the amplification of each sample. Duplicate reactions were combined and the PCR amplicons were purified using Agencourt AMPure XP Kit (Beckman Coulter, Inc., Brea, CA) according to the manufacturer's protocol. Amplicon pyrosequencing was performed using 454 Junior with GS FLX titanium chemistry (454 Life Science, Branford, CT) at the Department of Microbiology and Molecular Genetics, Michigan State University.

#### Sequence analysis

Sequences were analyzed using Mothur version 1.30 [66]. Readings with exact matches to the linker, barcode, spacer, and primer sequences were retained. The shhh.flows command, a re-implementation of the PyroNoise alogorithm (Quince et al., 2009), was used to denoise flowgrams. Sequences less than 200 bases or with homoploymers of 8 or more were removed. Sequences were aligned using the SILVA database, and chimeras were removed using Uchime (Edgar et al., 2011). Taxonomy was assigned using a cut-off score of 80% based on RDP training set 9, available within Mothur. The data set was then rarified to 2225 sequences per sample. The average neighbor-clustering algorithm was used to assign operational taxonomic units (OTUs) based on 97% similarity. Commands made available within Mothur were used to calculate the Bray-Curtis index and the Chao1 estimate and Shannon-Weiner diversity.

### Statistical analysis

Differences among aggregate fractions in intact and ground samples in terms of the soil properties, the cumulative CO_2_ emission during incubation, the amounts of leaf material lost during the incubation, and pore characteristics were analyzed using the PROC MIXED procedure of SAS 9.3 [67]. Differences among the fractions and ground/intact samples were declared to be statistically significant at p<0.05 level, while differences significant at p<0.1 level are mentioned as observed trends.

Compositions of soil microbial communities were summarized using Principal Coordinate Analysis (PCoA) applied to Bray-Curtis dissimilarity matrix [68], and conducted using R-package labdsv. Comparisons between Bray-Curtis dissimilarities of the intact and ground samples of different aggregate fractions were performed using Permutational Multivariate Analysis of Variance (PMAV) [69] that was conducted using R-package vegan. Comparisons between individual microbial groups in different aggregate fractions were conducted using Wilcox rank sum test.

## Results

### Soil pore characteristics

**Fig 1:**
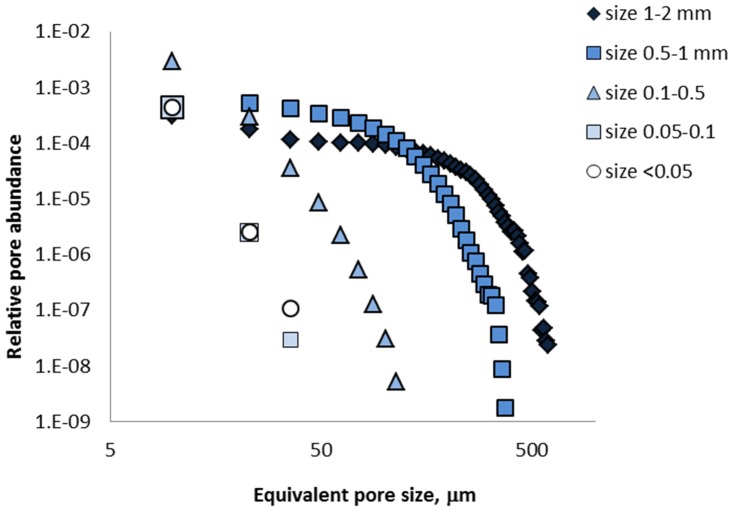
Average pore size distributions in the studied soil aggregate fractions. The relative abundance is calculated as a ratio between the numbers of pore medial axes voxels and the total image voxels.

As was intended, the samples created from the aggregate fractions of different sizes had highly contrasting pore size distributions (Fig 1 and Table 1). The biggest differences between the fractions were present in terms of image-based porosity values, maximum pore sizes, and pore connectivity. Maximum pore diameters were more than one order of magnitude larger in the 1–2 mm aggregate fraction than in the two smallest fractions (i.e. <0.05 and 0.05–0.1 mm). In the two largest aggregate fractions, 99% of the pore space visible on μCT images (>6.5 μm) was interconnected, while there were essentially no connections between >6.5 μm pores in the two smallest aggregate fractions. The two largest aggregate fractions had average pore diameters of 104 and 56 μm, respectively, while for the three smallest fractions the average pore diameter values were only 10–11 μm (Table 1).

**Table 1:** Summary of selected pore characteristics of the studied soil aggregate fractions.

Fraction size,	Total porosity,	Image-based porosity(> 6.5 μm),	Weighted average pore diameter,	Maximum pore diameter,	Pore space connectivity,
mm	cm^3^ cm^-3^	cm^3^ cm^-3^	μm	μm	%
**1.0–2.0**	0.562	0.405a*	104a	568a	99a
**0.5–1**	0.532	0.410a	56b	348b	99a
**0.1–0.5**	0.547	0.120b	11c	107c	60b
**0.05–0.1**	0.566	0.045c	10c	36d	<1c
**<0.05**	0.577	0.036c	10c	29d	<1c

*Means within the same column followed by the same letter are not significantly different from each other (p<0.05).

The average pore size of the 0.1–0.5 mm fraction was similar to that of the smallest two fractions (<0.05 and 0.05–0.1 mm), however the maximum size of the pores present in the 0.1–0.5 mm fraction was substantially larger than that in the smallest two (Table 1). Approximately 1/3 of the >6.5 μm pores in the 0.1–0.5 mm fraction were in the 35–100 μm size range, whereas no such pores were observed in the two smallest fractions (Fig 1). In the 0.1–0.5 mm fraction approximately 60% of the >6.5 μm pore space was interconnected.

Pore-size distributions of the ground samples were indistinguishable in their characteristics from those of the <0.05 mm fraction. The concentrations of C and N varied across the studied aggregate fractions and were the greatest in the 0.05–0.1 mm fraction (S2 Table).

### μ-CT leaf images

**Fig 2:**
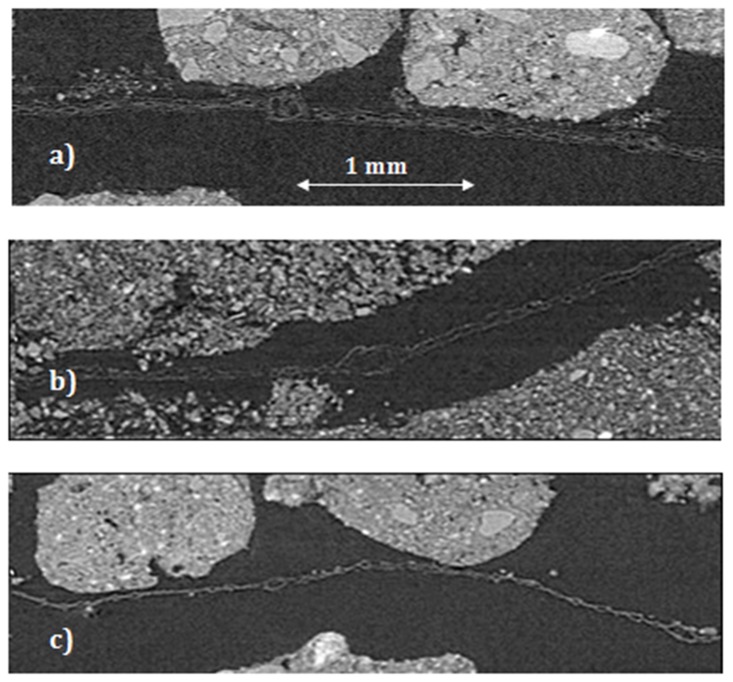
Examples of the leaf images from a) the leaf in a sample prior to incubation, and the leaves after 120 days of incubation in the samples of b) intact 0.05–0.1 mm aggregate fraction, and c) intact 1.0–2.0 mm aggregate fraction. Note that the large pores around the leaf in the 0.05–0.1 mm (b) have formed after the incubation when the samples were air-dried prior to the second scanning.

**Fig 3:**
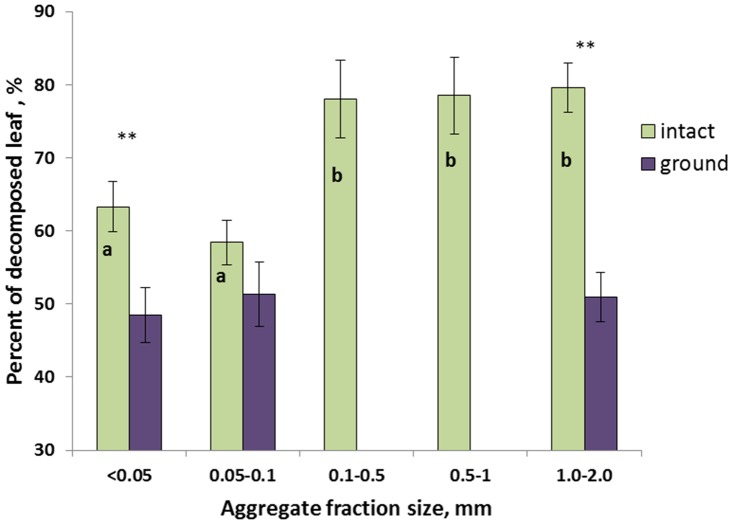
Percent of the corn leaves decomposed during the 120 day incubation as determined by X-ray μCT image analysis. Different letters within the intact group mark aggregate fractions significantly different from each other (p<0.05), while the differences among the aggregate fractions of the ground group were not statistically significant. The differences between the intact and ground aggregate fractions of <0.05 mm and 1.0–2.0 mm sizes were statistically significant (p<0.05) and are marked with **.

Corn leaves were clearly visible and well identified on the μCT-images (Fig 2). On average, the size of the original leaves as determined from the images was around 12 mega-voxels, while it decreased to 2 to 6 mega-voxels at the end of the 120 day incubation. Leaf decomposition was much stronger in the intact aggregate fractions of the three largest sizes, i.e., 0.1–0.5, 0.5–1.0, and 1.0–2.0 mm, as compared to the two smallest fractions (Fig 3). Approximately 75 to 80% of the original leaves were decomposed during the incubation in the three largest aggregate fractions, with the remaining leaf pieces estimated from the images of only 2–3 mega-voxels in size (Fig 2). Approximately 60% of the leaves were decomposed in the two smallest intact aggregate fractions with the remaining leaf pieces of 5–6 mega-voxels in size (Fig 2).

Leaf decomposition was the lowest in the ground samples. Only 50% of the leaves was found to be decomposed in the three aggregate size fractions that were subjected to scanning after the incubation, that is the fractions <0.05, 0.05–0.1, and 1–2 mm. Numerically, in all three fractions the amounts of leaf decomposed in the ground samples were lower than that in their respective intact counterparts; the differences were statistically significant for the <0.05 and 1–2 mm fractions (Fig 3).

### Carbon emission

**Fig 4:**
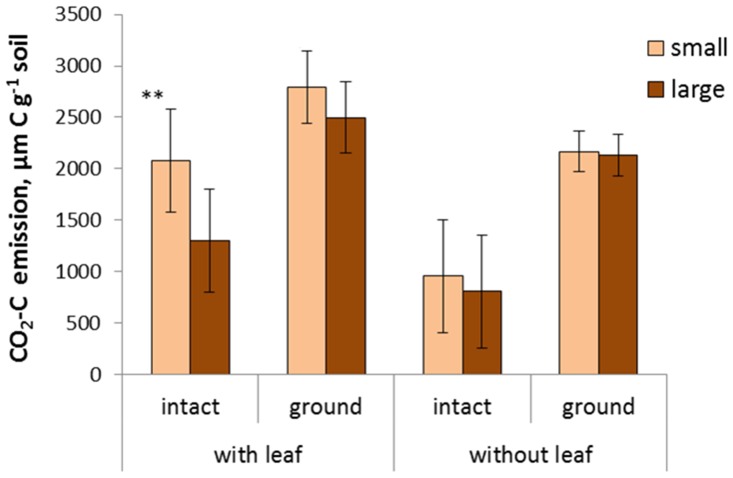
Cumulative amount of the CO_2_-C emitted from the small (<0.1 mm) and large (0.1–2 mm) fractions with and without corn leaf during the 120 day incubation in intact and ground samples. In all aggregate size fractions, both ground and intact, the CO_**2**_-C emitted from the samples with leaves was significantly higher than that from the samples without leaves (p<0.05). In all aggregate size fractions, both with and without leaf, the CO_**2**_-C emitted from the ground samples was significantly higher than that from the intact samples (p<0.05). The significant difference between the small and large aggregate fractions is marked with ** (p<0.05).

In all studied aggregate fractions the cumulative CO_2_ emitted during the 120 days of incubation from the ground samples was significantly higher than that in the intact samples across all aggregate fraction sizes with and without leaves (p<0.05). Expectedly, the cumulative CO_2_ from the samples with leaves was significantly higher than that in the samples without leaves (p<0.05) (Fig 4).

The samples from the two smallest aggregate fractions (<0.05 and 0.05–0.1 mm) behaved very similar to each other in terms of their total amounts of emitted CO_2_. Likewise, the samples from the three largest fractions (0.1–0.5, 0.5–1.0, and 1–2 mm) were very similar in their CO_2_ emissions. Thus for clarity in presenting the results we combined them into two groups that will be further referred to as small and large aggregate fractions, respectively. Numerically, the total CO_2_ emitted from the small aggregate fractions was higher than that from the large fractions in both ground and intact samples with and without leaves (Fig 4). However, only in the intact samples with leaves the difference between small and large fractions was statistically significant (p< 0.05).

**Fig 5:**
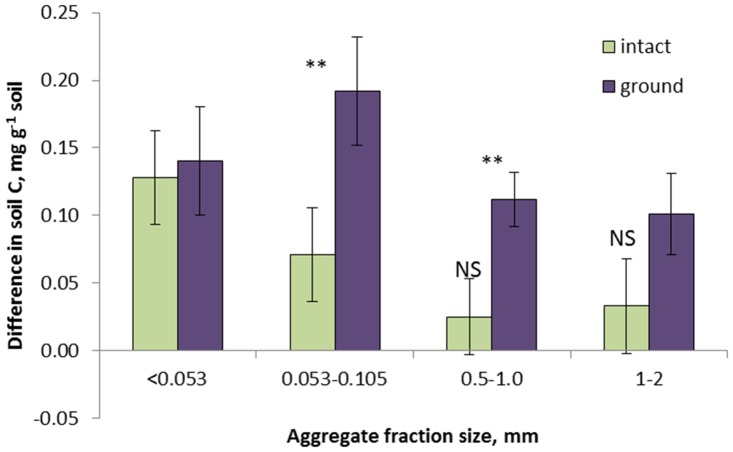
Differences between C levels in the soil layers adjacent to the corn leaf in the samples of the studied aggregate fractions after 120-day incubations and initial soil C content in the studied aggregate fractions (S3 Table). Cases when the intact and ground fractions are significantly different from each other are marked with ** (p<0.05). All differences except those marked with NS are significantly greater than zero (p<0.05).

After 120 day incubations the C level in the soil layer adjacent to the leaf increased substantially in the ground samples of all fraction sizes and in the intact samples of the two smallest fractions (Fig 5). The changes in the C content were very similar in the intact and ground samples of the smallest aggregate fraction. The changes were significantly lower in the intact samples as compared to the ground ones in the 0.05–0.1 and 0.5–1.0 mm fractions (p<0.05) and were numerically lower in the 1–2 mm fraction. The changes in C after incubation in the intact samples of the two largest fractions were not significantly different from zero.

### Microbial community composition on the incubated leaves and adjacent soil

**Fig 6:**
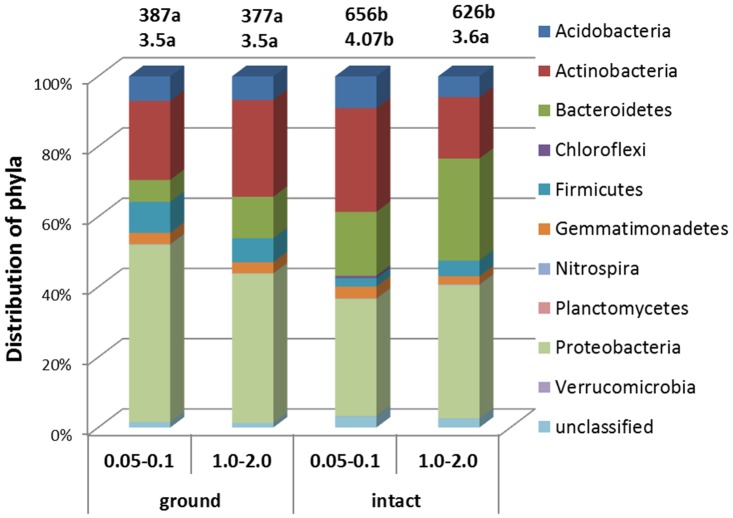
Distribution of the relative abundance of bacteria identified at the phylum level on decomposing corn leaves in intact and ground samples of 0.05–0.1 mm and 1–2 mm aggregate fractions after 14 day incubation. The numbers above the columns represent values of Chao (top) and Shannon (bottom) diversity indexes with different letters following each index indicating statistically significant differences among the four studied groups (p<0.05).

Bacteria belonging to 10 phyla were identified on the decomposing corn leaves from the studied samples (Fig 6). The most abundant were Proteobacteria with α-proteobacteria (*Afipia*, *Bradyrhizobium*, *Devosia*, *Phenylobacterium*, *Rhizobium*, *Roseomonas*, *Skermanella*), β-proteobacteria (*Burkholderia*, *Massilia*), and δ-proteobacteria (*Sorangium*) present, followed by Actibnobacteria (*Actinoallomurus*, *Actinomadura*, *Arthrobacter*, *Blastococcus*, *Dactylosporangium*, *Kribbella*, *Leifsonia*, *Nocardia*, *Nonomuraea*, *Pseudonocardia*, *Streptosporangium*, *Terrabacter*), and Bacteroidetes (*Adhaeribacter*, *Chitinophaga*, *Flavisolibacter*, *Niastella*, *Segetibacter*). Proteobacteria tended to be in greater abundance in ground samples, Actinobacteria tended to be less abundant in the intact samples of the 1–2 mm aggregate fraction, while Bacteroidetes were almost twice as abundant in the intact 1–2 mm aggregate fraction samples as in the 0.05–0.1 mm fraction and in the ground samples. The ground samples had significantly lower values of the Chao diversity indexes, while the intact 0.05–0.1 mm fraction had higher value of Shannon diversity index than both the ground samples and the intact 1–2 mm fraction (Fig 6).

**Fig 7:**
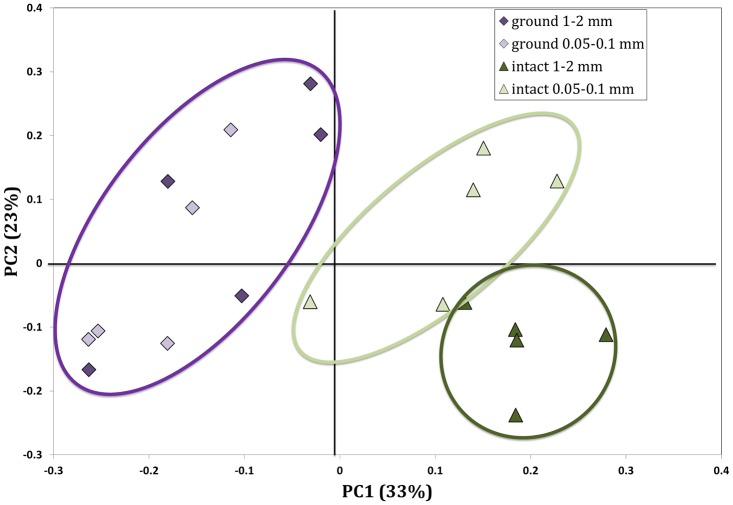
Principal coordinate analysis results based on the Bray-Curtis dissimilarities from the decomposing corn leaves in intact and ground samples of 0.05–0.1 mm and 1–2 mm aggregate fractions after 14 day incubation. Intact 0.05–0.1 and 1–2 mm fractions are significantly different from each other and from the ground fractions (p<0.05).

In Fig 7 we present results of PCoA based on Bray-Curtis dissimilarity of all detectable OTUs at the 97% similarity cutoff from phylogenetic composition of the bacterial communities as measured with next generation sequencing. The separation between the samples occurred primarily along the 1^st^ principal coordinate (PC1). The intact samples of the 1–2 mm fraction clustered separately from those of the intact 0.05–0.1 mm fraction, while the ground samples of the two fractions clustered together. PMAV revealed that in the intact samples bacterial community structure of the 1–2 mm fraction was significantly different from that of the 0.05–0.1 mm fraction (p<0.01), while in the ground samples community structures of the two studied fractions were not significantly different from each other (p<0.3).

We specifically focused on the individual OTUs the relative abundances of which significantly differed (p<0.05) between 1–2 mm and 0.05–0.1 mm fractions of the intact samples (Table 2). Such differences were observed in 21 out of top 100 most abundant OTUs. Fourteen of these groups were more abundant in the 0.05–0.1 mm fraction. Out of those, six belonged to *Acidobacteria*, namely, 5 to *Acidobacteria-Gp1* and 1 to *Acidobacteria-Gp6* classes. Among the remaining seven that were more abundant in the 1–2 mm fraction were two members of *Streptosporangiaceae* family, and members of *Niastella*, *Burkholderia*, *Afipia*, *Paenibacillus*, and *Nitrospira* genus.

**Table 2:** List of the bacteria OTUs (from the 100 most abundant ones) on decomposing corn leaves, the relative abundance of which was significantly different between the 1–2 mm aggregate fraction and the 0.05–0.1 mm aggregate fraction after 14 days of incubation (p<0.05).

Phylum	Class	Order	Family	Genus	Pore size comparisons
*Bacteroidetes*	*Sphingobacteria*	*Sphingobacteriales*	*Chitinophagaceae*	*Niastella*	L>S
*Proteobacteria*	*β-proteobacteria*	*Burkholderiales*	*Burkholderiaceae*	*Burkholderia*	L>S
*Proteobacteria*	*γ-proteobacteria*	*Xanthomonadales*	*Xanthomonadaceae*	*unclassified*	S>L
*Actinobacteria*	*Actinobacteria*	*Actinomycetales*	*Streptosporangiaceae*	*unclassified*	L>S
*Acidobacteria*	*Acidobacteria_Gp6*	*Gp6*	*unclassified*	*unclassified*	S>L
*Proteobacteria*	*α-proteobacteria*	*unclassified*	*unclassified*	*unclassified*	S>L
*Bacteroidetes*	*Sphingobacteria*	*Sphingobacteriales*	*Chitinophagaceae*	*Segetibacter*	S>L
*Proteobacteria*	*α-proteobacteria*	*Rhizobiales*	*Bradyrhizobiaceae*	*Afipia*	L>S
*Acidobacteria*	*Acidobacteria_Gp1*	*Gp1*	*unclassified*	*unclassified*	S>L
*Acidobacteria*	*Acidobacteria_Gp1*	*unclassified*	*unclassified*	*unclassified*	S>L
*Gemmatimonadetes*	*Gemmatimonadetes*	*Gemmatimonadales*	*Gemmatimonadaceae*	*Gemmatimonas*	S>L
*Actinobacteria*	*Actinobacteria*	*Actinomycetales*	*Streptosporangiaceae*	*Streptosporangium*	L>S
*Firmicutes*	*Bacilli*	*Bacillales*	*Paenibacillaceae_1*	*Paenibacillus*	L>S
*Actinobacteria*	*Actinobacteria*	*Actinomycetales*	*Nocardiaceae*	*unclassified*	S>L
*Chloroflexi*	*Ktedonobacteria*	*Ktedonobacterales*	*Thermosporotrichaceae*	*Thermosporothrix*	S>L
*Actinobacteria*	*Actinobacteria*	*Actinomycetales*	*Intrasporangiaceae*	*unclassified*	S>L
*Acidobacteria*	*Acidobacteria_Gp1*	*Gp1*	*unclassified*	*unclassified*	S>L
*Acidobacteria*	*Acidobacteria_Gp1*	*Gp1*	*unclassified*	*unclassified*	S>L
*Acidobacteria*	*Acidobacteria_Gp1*	*Gp1*	*unclassified*	*unclassified*	S>L
*Actinobacteria*	*Actinobacteria*	*Actinomycetales*	*Catenulisporaceae*	*Catenulispora*	S>L
*Nitrospira*	*Nitrospira*	*Nitrospirales*	*Nitrospiraceae*	*Nitrospira*	L>S

The cases when relative abundance was higher in the large fraction are marked by ‘L>S’ in the Pore size comparisons column, while the cases when relative abundance was higher in the small fraction are marked by ‘S>L’. The list is from most to least abundant OTUs.

## Discussion

### Effect of pore characteristics on plant residue decomposition

Decomposition of the added plant residue was substantially affected by the pore characteristics (Figs 2 and 3), being the greatest in the three largest fractions of the intact aggregates while substantially lower in the two smallest fractions. Note that mineralogical or chemical differences among the aggregate fractions could not be the cause for such a substantial difference between leaf decomposition in these two aggregate groups, therefore we attributed these differences to the differences in pore size distributions and pore connectivity. Indeed, when the samples were ground, the amount of the decomposed leaf in them remained consistently similar, i.e., around 50%, regardless of what aggregate fraction they originated from (Fig 3). Since ground samples were identical in their chemical composition and mineralogy to their intact aggregate fraction counterparts, we can safely conclude that the differences in pore characteristics among the intact aggregate fraction samples are the most likely cause for the differences in residue decomposition observed in this study.

Previous studies provide an ancillary support of our finding that pore characteristics influence decomposition of newly added organic substrate [70–72]. For example, Haling et al. [71] observed slower decomposition of plant roots added to the soil samples in the samples with greater bulk density, that is, with lower total porosity. Juarez et al. [72] showed that greater mineralization of added fructose occurred in intact as opposed to sieved and dispersed soil samples, that is, in the samples with substantial presence of highly connected large (>32 μm) pores. However, to the best of our knowledge, our study is the first report of the influence of pore characteristics on the decomposition of added plant residues. It is the utilization of the novel X-ray μCT tools that enabled us to accurately estimate the quantities of plant residue which remained undecomposed in the soil samples after prolonged incubation.

One pore characteristic that separates the three largest aggregate factions from the rest is the presence of medium sized pores (>30–40 μm). Such pores were completely absent in the smallest two fractions and in the ground samples, while they were in sizeable presence and were substantially interconnected in all three largest fractions (Fig 1 and Table 1). Importance of medium sized pores for enhanced decomposition of both added and native organic C in soil has been noted before [28, 30, 73]; and also greater presence of such pores was found to be associated with lower native organic C in soil aggregates [74].

**Fig 8:**
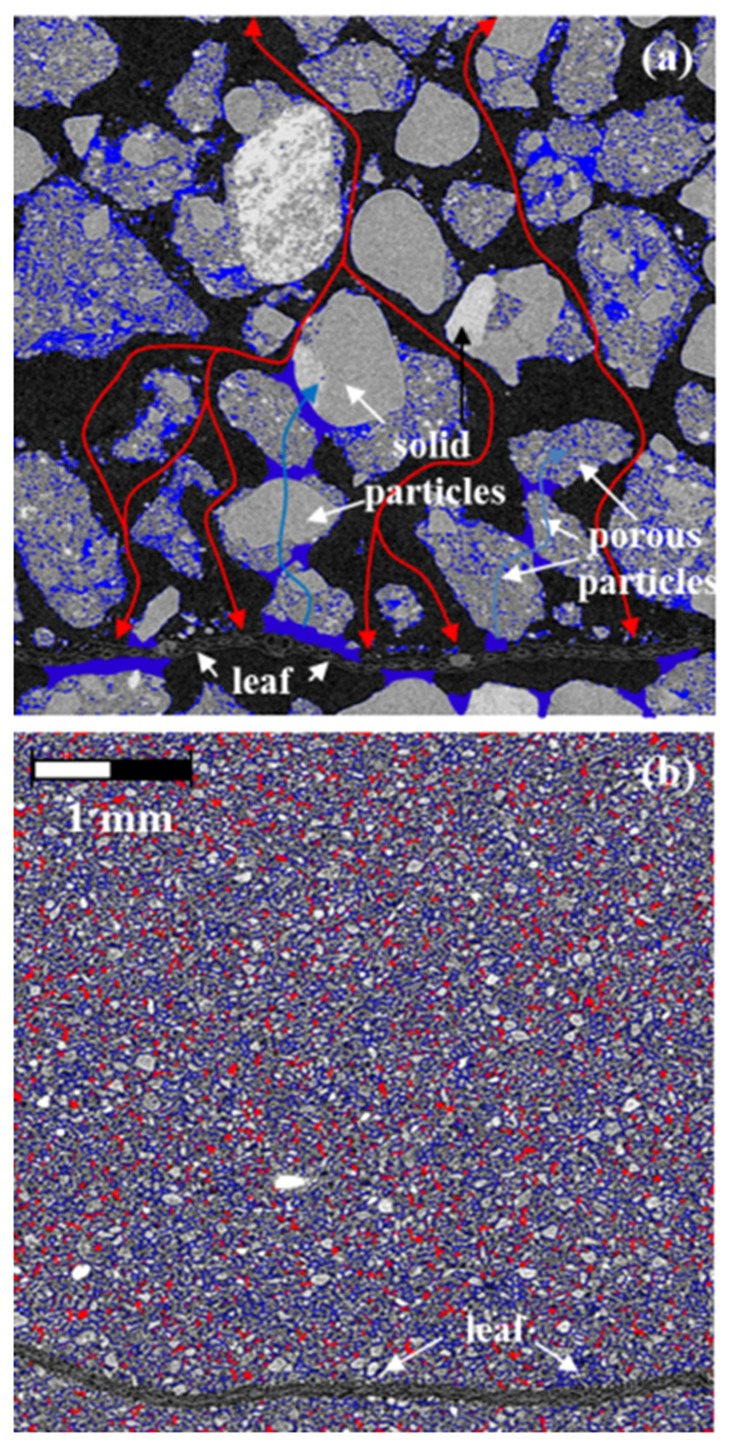
Conceptual representation of pores with different functional properties on images of intact aggregate fractions of 1.0–2.0 mm (a) and 0.05–0.1 mm (b) at 50% water filled porosity. Schematic locations of water menisci between the soil particles in the 1.0–2.0 mm aggregate fractions and pores likely filled with water are marked with blue. Pores likely filled with air in the 0.05–0.1 mm fraction are marked with red, while possible air passes in the 1.0–2.0 mm fraction are marked with red arrows.

Presence of large interconnected pores substantially affected aeration conditions within the samples and greatly facilitated gaseous exchange between samples and atmosphere (Fig 8). The results of gas diffusion ratio computation using (Eq 1) showed that *D*_*p*_ */D*_*o*_ values ranged from 0.018 to 0.034 in the intact 1–2 mm aggregate fractions, while they ranged from 0.002 to 0.004 in the aggregate fractions <0.1 mm. This constitutes an almost 9-fold difference in the rates of air diffusion between samples with and without large pores.

Water distribution in the intact samples of the large aggregate fraction was highly non-uniform with most water being stored inside of the aggregates and in thin films between them; while large pores performed as preferential air conduits (Fig 8a). Abundance of small relatively uniformly distributed pores in the small aggregate fractions resulted in relatively uniform distribution of water (Fig 8b). However, water bridges between soil particles likely blocked gas transport pathways [75] thus limiting gas diffusion (Fig 8b). High gas diffusion in the large aggregate fractions likely resulted in better O_2_ supply to the leaves thus contributing to their more complete decomposition followed by the rapid escape of the produced CO_2_.

### Effect of pore characteristics on CO_2_ emission and soil C changes

The magnitude of the pore characteristics’ influence on the total amount of CO_2_ emitted from the samples during the incubation depended on the presence of plant residue. Even though the studied aggregate fractions were markedly different in their pore size distributions (Fig 1), in the samples without added plant residue there was no effect of pore characteristics on the overall amount of the native SOM decomposed during the incubation (Fig 4). This result is consistent with multiple previous reports of no differences in native C mineralization in soil samples where pore size distributions were modified by applying either sieving, dispersion, or different levels of soil compaction [31, 32, 68, 76, 77].

The increase in the CO_2_ emission after aggregate grinding was ultimately caused by the release of soil C previously not accessible to microorganisms indicating presence of physical protection ([28, 78–80]. It was essentially the same for all aggregate fractions. Therefore the absence of pore size influence on CO_2_ emission in intact samples without plant residue indicates that the main limitation was the lack of access of the decomposers to C sources rather than absence of optimal conditions for C decomposition.

In the samples with plant residue, substantially higher total amounts of CO_2_ were emitted during the incubations from the samples with small pores as opposed to the samples with large pores (Fig 4). However, we would like to exercise caution in attributing this difference primarily to the differences in pore characteristics. A tendency for higher CO_2_ emission in small as opposed to large aggregate fractions was observed in ground samples as well, even though the magnitude of the difference in the ground samples was almost three times smaller than that in the intact samples.

The differences in the amounts of emitted CO_2_ (Fig 4) along with the differences in the levels of C in soil adjacent to the leaves after the incubation (Fig 5) suggest that pore characteristics define the fate of the plant residue decomposition products. Plant residue decomposition starts with production of dissolved organic matter with particularly hydrophilic characteristics [81]. A rapid enrichment of the soil adjacent to the residue with decomposition products then follows; and decomposition products can move as far as 5 mm into the adjacent soil [82, 83]. In our study soluble decomposition products are expected to be easily diffused in water saturated fine pores of the ground samples and the samples of the smallest aggregate fractions, where high abundance of small pores promotes its diffusion into adjacent soil (Fig 8b). There we observed a substantial increase in C in the soil layer adjacent to the corn leaf (Fig 5). In the intact samples of large aggregate fractions the abundance of fine pores is lower and they are primarily located within the large aggregates separated from the fine pores in other aggregates and from the leaves by large inter-aggregate pores (Fig 8a). This considerably reduces the diffusion of the soluble decomposition products from the leaf surfaces into adjacent soil, thus resulting in a non-significant change in soil C in the layer adjacent to the leaf. However, greater gas diffusion creates local conditions for more complete *in situ* decomposition of plant residue material (Fig 8a).

Consistent with multiple reports, e.g., [39], it can be expected that upon entering soil the dissolved intermediate products of plant residue decomposition will stimulate decomposition of the native SOM, thus resulting in positive priming. The greater the diffusion of the plant decomposition products the greater priming effect can be expected. Current study does not allow us the separation of the CO_2_ originated from the native soil C and that originated from the added plant residue; a condition necessary to identify the presence of and to evaluate the magnitude of the priming effect. However, comparisons of the differences between the CO_2_ emitted in the samples with and without leaves with the amount of decomposed plant residues enable us to presume the possibility of priming taking place in the intact samples of the two smallest aggregate fractions. Specifically, in the intact samples of the three largest aggregate fractions the differences between the CO_2_ emitted in the samples with and without leaves is comparable to the amount of C in the decomposed leaf residue (Fig 4). However, in the intact samples of the two smallest sizes the difference is markedly greater than the amount of C in the decomposed leaf residue, with priming effect being the most likely explanation. These considerations support the suggestion made previously by Ruamps et al. [25] that distributions and configurations of soil pores can be a factor influencing presence and magnitude of priming effect.

### Effect of pore characteristics on microbial community composition

Abundance of Proteobacteria, Actinobacteria, and Firmicutes on the decomposing corn leaves was consistent with a number of studies demonstrating that many of the members of these groups are active decomposers of cellulose, including corn residues [84–87]. Specifically, *Arthrobacter*, *Bacillus*, *Blastococcus*, *Paenibacillus*, and *Rhizobium* groups were identified among active decomposes of corn residue in soil incubation studies [87].

We realize that grinding can have a substantial effect on microbial communities [77, 88] and is likely the cause for substantially lower values of Chao diversity indexes in the ground samples of this study. But it appears that soil material of both fractions responded to grinding by very similar compositions of microbial communities appearing after the 14 days of incubation.

Our results indicated substantial influence of pore characteristics on compositions of bacterial communities on the decomposing corn leaves (Fig 7, Table 2). These differences were not confounded by chemical and mineralogical differences between the two studied aggregate fractions, as microbial community compositions of the ground samples of both fractions did not differ in terms of their Bray-Curtis distances or abundances of specific microbial groups. Also note that soil moisture contents were the same in all samples, i.e. 50% of the total pore space was filled with water, as well as there were no differences in the total porosities among the studied aggregate fractions (Table 1). Thus, it was neither the overall amount of water present in the samples nor their total porosity, but the pore structure and the location of water and air conduits within the pores that influenced the observed differences in the bacterial communities on the plant residue.

A generally accepted concept of the mechanisms behind positive priming states that priming is an outcome of high activity of copiotrophic organisms decomposing plant residue and stimulating oligotrophic soil communities which, in their turn, contribute to greater decomposition of the native SOM [89]. Greater amounts of emitted CO_2_ along with lower amounts of decomposed plant residue in the 0.05–0.1 mm aggregate fraction and lower CO_2_ emissions along with higher amounts of decomposed plant residue in the 1–2 mm aggregate fraction in conjunction with the microbial community results in our study appear to be consistent with this mechanism. In the samples with small pores we observed both greater presence of Acidobacteria, a group regarded as oligotrophs commonly present in soils and involved in decomposition of native SOM [86, 90, 91], and still a substantial abundance of a number of Actinobacteria and Proteobacteria organisms, the groups which are regarded as copiotrophic. The samples with large pores were greater in abundances of only the groups known to be cellulose decomposers, i.e., Bacteroidetes, Proteobacteria, and Actinobacteria; also the groups particularly competitive in decomposition of more recalcitrant plant residues, i.e., Firmicutes; and those specifically found as active decomposers of corn residue in stable isotope probing studies, i.e., *Paenibacillus* [86, 87].

It was reported that differences in the degradability characteristics of plant residue can influence composition and dynamics of microbial communities involved in decomposition of the residue and associated decomposition of the native SOM [86]. It is also known that differences in microbial community composition on the decomposing plant residue can be an important factor influencing magnitude and rates of the decomposition process [92]. Moreover, microbial community composition inside macro-aggregates of the studied soil can be related to presence and characteristics of the intra-aggregate pores [61]. The results of this study indicate that differences in the environmental conditions in the soil surrounding the plant residue can also substantially influence microbial community composition. Still further research is needed to explore the specific mechanisms by which pore characteristics influence the interactions between decomposition of added plant residue, native SOM, and the involved microorganisms.

## Conclusions

The results of this study demonstrated that soil pore structure greatly influences the magnitude of decomposition of the added plant residue, likely simultaneously affecting the decomposition of the native SOM. Substantially higher amounts of plant residue were decomposed in the samples that had large (>30 μm) pores and high connectivity of the pores >6.5 μm. However, higher total amounts of CO_2_ were emitted from the samples with only small pores and with very low >6.5 μm pore connectivity. Moreover, an increase in C in the soil adjacent to the decomposing plant residue was also substantially higher in the small pore/low connectivity samples. Greater air diffusion and lower water diffusion in presence of large air-filled pores while greater water diffusion and limited air-diffusion in the samples with prevalent small pores are the likely reasons for the observed differences.

In absence of plant residue the influence of pore characteristics on the decomposition of the native SOM was found to be minor to nonexistent. This indicated that in the absence of plant residue the main limitation in decomposition of native SOM was the lack of access of the decomposers to C sources rather than absence of optimal conditions for C decomposition.

Microbial communities on decomposing plant residues in the samples with both large and small pores were dominated by copiotrophic organisms, many of which were known active decomposers of cellulose. However, a number of oligotrophic Acidobacteria groups were more abundant on the plant residue from the samples with small pores and low pore connectivity. As far as we know, this is the first report demonstrating that differences in soil micro-environmental conditions, i.e., pore characteristics, can substantially influence the structure of the microbial communities on the decomposing plant residue.

## Supporting Information

S1 Fig Schematic representation of the steps involved in setting up the incubation experiment for samples amended with corn leaf residue. (TIF)

S1 Table Summary of the numbers of replicated samples processed in the experiments of the study. (DOCX)

S2 Table Selected characteristics of the studied soil aggregate fractions. (DOCX)
