# Supplementary figures and images for: Properties of Soil Pore Space Regulate Pathways of Plant Residue Decomposition and Community Structure of Associated Bacteria
Source: PLoS One. 2015 Apr 24;10(4):e0123999. doi: 10.1371/journal.pone.0123999 (PMC4409378; doi:10.1371/journal.pone.0123999)

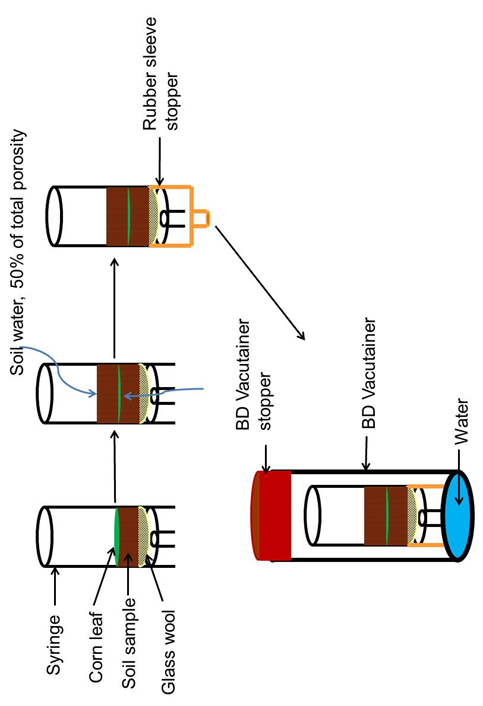

Supplement: S1 Fig — (TIF) [file pone.0123999.s001.tif]
