# Supplementary material for: Properties of Soil Pore Space Regulate Pathways of Plant Residue Decomposition and Community Structure of Associated Bacteria
Source: PLoS One. 2015 Apr 24;10(4):e0123999. doi: 10.1371/journal.pone.0123999 (PMC4409378; doi:10.1371/journal.pone.0123999)
Supplement: S1 Table — (DOCX) [file pone.0123999.s002.docx]

**Table S1. Summary of the numbers of replicated samples processed in the experiments of the study.** The first number in each cell corresponds to the samples with leaves and the second number corresponds to the samples without leaves.

|  |  |  | **Aggregate fraction size, mm** | | | | |
| --- | --- | --- | --- | --- | --- | --- | --- |
| **Sample type** | **Experiment** |  | **<0.05** | **0.05-0.10** | **0.10-0.50** | **0.50-1.00** | **1.00-2.00** |
| **Intact** | **Incubation** |  | 6/4 | 6/4 | 6/4 | 6/4 | 6/4 |
|  | **µCT*** |  | 6/- | 6/- | 2/- | 2/- | 6/- |
|  | **MCA*** |  | - | 5/- | - | - | 5/- |
|  |  |  |  |  |  |  |  |
| **Ground** | **Incubation** |  | 5/5 | 5/5 | 5/5 | 5/5 | 5/5 |
|  | **µCT** |  | 5/- | 5/- | - | - | 5/- |
|  | **MCA** |  | - | 5/- | - | - | 5/- |

* µCT – X-ray computed micro-tomography scanning

* MCA – Microbial community analysis
