# Supplementary material for: Properties of Soil Pore Space Regulate Pathways of Plant Residue Decomposition and Community Structure of Associated Bacteria
Source: PLoS One. 2015 Apr 24;10(4):e0123999. doi: 10.1371/journal.pone.0123999 (PMC4409378; doi:10.1371/journal.pone.0123999)
Supplement: S2 Table — (DOCX) [file pone.0123999.s003.docx]

**Table S2. Selected characteristics of the studied soil aggregate fractions.**

| **Fraction**  **size** | **pH** | **TC** | **TN** |
| --- | --- | --- | --- |
| mm |  | mg g^-1^ soil | |
| **<0.05** | 6.1 | 10.0b | 0.46a |
| **0.05-0.1** | 6.0 | 11.5a | 0.51a |
| **0.1-0.5** | 6.0 | 9.0c | 0.39b |
| **0.5-1.0** | 6.1 | 7.8e | 0.33c |
| **1.0-2.0** | 6.1 | 8.3d | 0.36c |

* Means within the same column followed by the same letter are not significantly different from each other (p<0.05).

TC: total carbon; TN: total nitrogen
